# Efficient conversion of acetate into lipids by the oleaginous yeast *Cryptococcus curvatus*

**DOI:** 10.1186/s13068-015-0371-3

**Published:** 2015-11-25

**Authors:** Zhiwei Gong, Hongwei Shen, Wengting Zhou, Yandan Wang, Xiaobing Yang, Zongbao K. Zhao

**Affiliations:** College of Chemical Engineering and Technology, Wuhan University of Science and Technology, 947 Heping Road, Wuhan, 430081 People’s Republic of China; Dalian National Laboratory for Clean Energy and Dalian Institute of Chemical Physics, CAS, 457 Zhongshan Road, Dalian, 116023 People’s Republic of China

**Keywords:** Oleaginous yeast, *Cryptococcus curvatus*, Acetic acid, Continuous culture, Microbial lipids, Biodiesel, Biomass hydrolysates

## Abstract

**Background:**

Acetic acid is routinely generated during lignocelluloses degradation, syngas fermentation, dark hydrogen fermentation and other anaerobic bioprocesses. Acetate stream is commonly regarded as a by-product and detrimental to microbial cell growth. Conversion of acetate into lipids by oleaginous yeasts may be a good choice to turn the by-product into treasure.

**Results:**

Ten well-known oleaginous yeasts were evaluated for lipid production on acetate under flask culture conditions. It was found that all of those yeasts could use acetate for microbial lipid production. In particular, *Cryptococcus curvatus* accumulated lipids up to 73.4 % of its dry cell mass weight. When the culture was held in a 3-L stirred-tank bioreactor, cell mass, lipid content, lipid yield and acetate consumption rate were 8.1 g/L, 49.9 %, 0.15 g/g and 0.64 g/L/h, respectively. The fatty acid compositional profiles of the acetate-derived lipids were similar to those of vegetable oil, suggesting their potential for biodiesel production. Continuous cultivation of *C. curvatus* was conducted under nitrogen-rich condition at a dilution rate of 0.04 h^−1^, the maximal lipid content and lipid yield were 56.7 % and 0.18 g/g, respectively. The specific lipid formation rate, lipid content and lipid yield were all higher under nitrogen-rich conditions than those obtained under nitrogen-limited conditions at the same dilution rates. Effective lipid production by *C. curvatus* was observed on corn stover hydrolysates containing 15.9 g/L acetate.

**Conclusions:**

Acetate is an effective carbon source for microbial lipid production by oleaginous yeasts. Continuous cultivation of *C. curvatus* on acetate was promising for lipid production under both nitrogen-rich and nitrogen-limited conditions. These results provide valuable information for developing and designing more efficient acetate-into-lipids bioprocess.

## Background

Microbial lipids generated from low-cost substrates are potential alternative feedstock for biodiesel and oleochemical industries [1, 2]. Some oleaginous yeasts can accumulate lipids up to 70 % of their dry cell weight [3]. When sugars and related materials are used as substrates, lipid biosynthesis is often triggered by limitation of macronutrients, such as nitrogen or phosphate [4, 5].

Acetate is routinely present in aqueous stream of various biological processes but commonly recognized as a by-product. For example, because hemicelluloses are generally acetylated [6], biomass hydrolysates contain acetate [7]. In addition, acetate is co-produced during syngas fermentation, dark hydrogen fermentation and other anaerobic bioprocesses [8–11]. Recently, *Acetobacterium woodii* has been reported to produce 51 g/L of acetate from CO_2_/H_2_ [12]. Acetate has been known as an inhibitor for cell growth of some oleaginous microorganisms [7, 13, 14].

It is worth mentioning that acetate has been proven toxic to cell growth but beneficial to lipid accumulation by the yeast *Rhodosporidium toruloides* Y4 [15]. It is conceivable that acetate can be assimilated and converted into acetyl-CoA, the precursor to lipid biosynthesis [3, 16]. The metabolic scheme and associated stoichiometric equations have already been established previously [17]. More recently, acetate has been explored as substrate for the cultivation of oleaginous species [10, 11, 18–21]. Volatile fatty acids (mixtures of acetate, propionate and butyrate) at a low concentration of 2 g/L were sufficient to support *Cryptococcus albidus* cells for lipid production and lipid yield reached 0.167 g/g [18]. Because *Cryptococcus curvatus* and *Yarrowia lipolytica* exhibited poor cell growth on acetate, a two-stage culture process, cell proliferation on glucose and lipid biosynthesis on acetate, was developed. Lipid content and lipid yield for *C. curvatus* cells were 50 % and 0.15 g/g, and for *Y. lipolytica*, 40.7 % and 0.13 g/g, respectively [19, 20]. *C. curvatus* has been confirmed to grow better under neutral condition than acidic condition when acetate is the sole carbon source. A pH-stat culture fed with pure acetate was then established. Cell mass and lipid content reached 168 g/L and 75.0 %, respectively [10]. However, fed-batch culture on acetate might not be viable because acetate is usually present in aqueous stream of various biological processes at relatively low concentrations.

Continuous culture has been regarded as a promising strategy for lipid production [22–25]. When a stream containing acetate at low concentration is considered as feedstock, continuous culture may be more appropriate for lipid production. Previously, continuous cultivation of *C. curvatus* cells on hydrogen production effluent containing acetate has been investigated, and cellular lipid content was only 13.5 % [10]. To further explore the potential of lipid production on acetate, here we screened ten well-known oleaginous yeasts under flask culture conditions, and identified *C. curvatus* as a superior strain for such purpose. Continuous cultures under nitrogen-rich or nitrogen-limited conditions were evaluated, and the results provided useful information for developing and designing more efficient acetate-into-lipids bioprocess.

## Results and discussion

### Batch culture for lipid production on acetate

Short chain organic acids, especially acetate, have been reported suitable for lipid production by several oleaginous yeasts [10, 11, 18, 20, 21]. However, the capacities of lipid production on acetate have not been systematically compared and evaluated. In this study, ten well-known oleaginous yeasts were evaluated for lipid production using acetate as sole carbon source, and the results are shown in Table 1. It was clear that all of these oleaginous yeasts over-produced lipids. Among them, *Trichosporon cutaneum* AS 2.571, *Trichosporon fermentans* CICC 1368, *C. curvatus* ATCC 20509 and *R. toruloides* Y4 achieved lipid contents more than 50 % of their dry cell weight when the cultures were terminated after 72 h. The highest cell mass and lipid titre were 7.5 and 4.4 g/L, respectively, by *T. cutaneum*, and the highest lipid content reached 73.4 % by *C. curvatus*.

**Table 1:** Results of lipid production on acetate by oleaginous yeasts

Strain	ΔpH^a^	Cell mass (g/L)	Lipid (g/L)	Lipid content (%)
*L. starkeyi* AS 2.1560	2.07 ± 0.02	3.2 ± 0.2	0.6 ± 0.1	17.1 ± 1.4
*Y. lipolytica* AS 2.1398	2.10 ± 0.01	4.1 ± 0.5	0.5 ± 0.0	12.2 ± 0.4
*T. cutaneum* AS 2.571	2.40 ± 0.02	7.5 ± 0.1	4.4 ± 0.0	58.5 ± 0.5
*T. fermentans* CICC 1368	2.35 ± 0.02	6.8 ± 0.5	3.8 ± 0.3	55.4 ± 0.8
*R. glutinis* AS 2.107	2.26 ± 0.02	2.5 ± 0.1	0.7 ± 0.1	27.0 ± 2.1
*R. mucilaginosa* AS 2.1515	2.03 ± 0.04	2.6 ± 0.2	0.6 ± 0.1	21.8 ± 0.6
*R. minuta* AS 2.277	2.02 ± 0.01	1.8 ± 0.1	0.5 ± 0.0	30.2 ± 0.3
*C. curvatus* ATCC 20509	2.38 ± 0.01	5.7 ± 0.1	4.2 ± 0.1	73.4 ± 0.6
*R. toruloides* ATCC 10788	2.26 ± 0.03	1.2 ± 0.0	0.4 ± 0.0	33.0 ± 3.0
*R. toruloides* Y4	2.38 ± 0.02	2.6 ± 0.3	1.5 ± 0.2	54.9 ± 1.3

^a^Delta pH (ΔpH) was obtained by subtracting the initial pH value from the final pH value

**Fig. 1:**
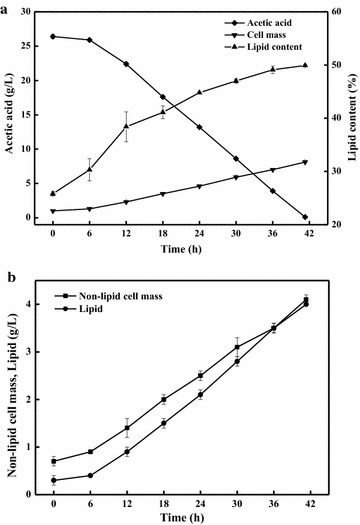
Results of lipid production by *C. curvatus* on acetate. **a** Profiles of acetate consumption, cell growth and lipid accumulation. **b** The evolution of lipid and non-lipid cell mass

Optimal culture pH range was between 5.0 and 6.0 during lipid production from sugars by most oleaginous yeasts [26, 27]. However, all these yeasts grew unsuccessfully in acetate assimilation medium when the initial pH was set at 5.5 (data not shown). Because acetate has a p*K*a of 4.75, at acidic pH, acetate appears largely in undissociated form, which imposes inhibitory effects on cell proliferation; however, acetic acid in its dissociated form is much less toxic [6]. When the culture pH was at 5.5, about 15 % acetic acid was in the undissociated form, which exerted inhibitory effect on cell growth. When the cultures were at pH 7.0, 99 % acetic acid was dissociated into acetate anion. It was found that culture pH increased over time to above 9.0 at the end of the culture, indicating the consumption of acetate in the medium. Therefore, a nitrogen-limited batch culture of *C. curvatus* was performed in a 3-L stirred-tank bioreactor to keep pH constant. Culture pH, temperature, agitation and aeration rate were set at 7.0, 30 °C, 500 rpm and 0.8 vvm, respectively. Our results indicated that 26.4 g/L of acetate was consumed within 41.3 h (Fig. 1a), corresponding to an acetate consumption rate of 0.64 g/L/h. Cell mass, lipid content, lipid yield and lipid productivity were 8.1 g/L, 49.9 %, 0.15 g/g and 2.32 g/L/d, respectively. The lipid content and lipid yield were similar to those obtained by *C. curvatus* under two-stage fed-batch culture conditions [19]. It was also found that non-lipid cell mass increased continuously (Fig. 1b), which was different from the general trend that oleaginous yeasts favored lipid biosynthesis rather than cell proliferation under nitrogen-limited conditions [4]. In fact, *Cryptococcus terricola* was the only reported oleaginous yeast that produced lipids in growth phase, albeit on glucose [28].

Microbial lipids produced from acetate by *C. curvatus* were transmethylated and fatty acid compositional profiles were analyzed by gas chromatography. It was found that the sample consisted mainly of long-chain fatty acids with 16 and 18 carbon atoms, including 0.9 % myristic acid, 32.0 % palmitic acid, 0.4 % palmitoleic acid, 23.6 % stearic acid, 39.5 % oleic acid and 0.2 % linoleic acid. Specifically, palmitic acid, stearic acid and oleic acid together accounted for over 95 % of the total fatty acids, which were similar to the fatty acid compositional profiles of lipids produced from lignocellulosic sugars by the same yeast [27, 29]. Such compositional profiles were also comparable to those of vegetable oil, suggesting that acetate-derived lipids could be explored for biodiesel production [30].

### Continuous cultivation of *C. curvatus* on acetate under nitrogen-rich conditions

**Fig. 2:**
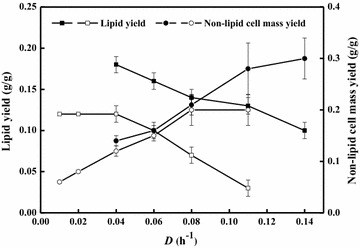
Steady-state lipid yields and non-lipid cell mass yields of *C. curvatus* at different dilution rates under nitrogen-rich and nitrogen-limited conditions. *Error bars* mean ± standard deviation of four samples

Acetate was evaluated for growing *C. curvatus* cells in nitrogen-rich medium under continuous culture conditions with dilution rates ranged from 0.04 to 0.14 h^−1^. Surprisingly, lipid contents were higher than 20 %, and lipid content decreased from 56.7 % at a dilution rate of 0.04 h^−1^ to 25.5 % at a dilution rate of 0.14 h^−1^ (Table 2). Similarly, lipid yield also dropped from 0.18 to 0.10 g/g (Fig. 2). The fact that a lipid yield of 0.18 g/g was achieved suggested that the majority of acetate was channeled into lipid biosynthesis. However, the C/N ratio of the feeding medium was 1.76, which was substantially lower than those used for microbial lipid production on sugars and related materials [31, 32]. The lipid productivity increased when the dilution rate decreased (Table 2). The highest lipid productivity was 0.73 g/L/d at the dilution rate of 0.04 h^−1^, which was significantly lower than that by batch culture. An early study found that ammonia inhibited cell growth and lipid biosynthesis when *C. curvatus* was cultivated on acetate at pH above 7.5, likely due to the formation of excess intracellular ammonia that inhibited acyl-CoA synthase activity [33]. In the current study at pH 7.0 in the presence of 5.0 g/L NH_4_Cl, it seemed ammonium had little inhibitory effects on lipid accumulation. It should also be emphasized that the lipid contents of *R. toruloides* were below 10 % at various dilution rates under carbon-limited conditions [25], which was dramatically different from those of *C. curvatus*.

**Table 2:** Results of continuous cultivation of *C. curvatus* at various dilution rates under nitrogen-rich conditions

*D* (h^−1^)	Acetate consumption (g/L)	Residual acetate (g/L)	Cell mass (g/L)	Non-lipid cell mass (g/L)	Lipid (g/L)	Lipid content (%)	lipid yield (g/g)	Lipid productivity (g/L/d)
0.04	4.18 ± 0.02	0.67 ± 0.02	1.34 ± 0.03	0.58 ± 0.04	0.76 ± 0.03	56.71 ± 2.12	0.18 ± 0.01	0.73 ± 0.03
0.06	2.65 ± 0.07	2.20 ± 0.07	0.84 ± 0.02	0.42 ± 0.01	0.42 ± 0.02	50.29 ± 1.43	0.16 ± 0.01	0.60 ± 0.02
0.08	1.88 ± 0.07	2.97 ± 0.09	0.66 ± 0.02	0.39 ± 0.02	0.27 ± 0.01	41.17 ± 1.10	0.15 ± 0.01	0.52 ± 0.01
0.11	0.88 ± 0.06	3.97 ± 0.04	0.36 ± 0.03	0.25 ± 0.03	0.11 ± 0.01	31.06 ± 2.49	0.13 ± 0.01	0.29 ± 0.01
0.14	0.71 ± 0.07	4.14 ± 0.07	0.29 ± 0.02	0.21 ± 0.02	0.07 ± 0.01	25.50 ± 1.02	0.10 ± 0.01	0.24 ± 0.01

When extra yeast extract (0.4 g/L) and peptone (0.4 g/L) were added to the nitrogen-rich acetate medium, the C/N ratio slightly decreased to 1.67. However, lipid content dropped dramatically to below 20 % at a dilution rate of 0.08 h^−1^, this was because organic nitrogen sources facilitated cell growth by supplementing amino acids and related components. On the other hand, inorganic nitrogen sources might be less efficient in terms of supporting cell growth on acetate.

The non-lipid cell mass yield increased over the entire dilution rate ranges and reached 0.30 g/g at a dilution rate of 0.14 h^−1^ (Fig. 2), indicating that more acetate was used for cell growth at higher dilution rates. This was likely due that organic nitrogen sources were consumed rapidly for cell growth and thus limited the production of non-lipid cell mass under conditions with lower dilution rates. The maximal cell mass yield of 0.41 g/g was obtained at a dilution rate of 0.11 h^−1^, which was comparable to that of *R. toruloides* under carbon-limited condition using glucose as sole carbon source [25].

**Fig. 3:**
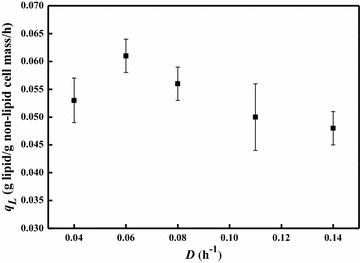
Steady-state specific lipid formation rates of *C. curvatus* at different dilution rates under nitrogen-rich conditions. *Error bars* mean ± standard deviation of four samples

The relationship between specific lipid formation rate and dilution rate is shown in Fig. 3. The specific lipid formation rate increased along with the dilution rate from 0.04 to 0.06 h^−1^, but then dropped when dilution rate increased further. The maximal specific lipid formation rate of 0.061 g/g non-lipid cell mass/h was observed at the dilution rate 0.06 h^−1^, which was even higher than that obtained by *R. toruloides* on glucose under the nitrogen-limited condition [25]. As lipid accumulation on sugars and related substrates by oleaginous yeasts is normally triggered by nitrogen starvation, it is important to remove excess nitrogen from nitrogen-rich substrates [4]. Interestingly, our results demonstrated that lipid production on acetate by *C. curvatus* could be operated under nitrogen-rich conditions, providing new opportunity to use acetate-containing wastes rich in ammonia nitrogen for microbial lipid production [10].

### Continuous cultivation of *C. curvatus* under nitrogen-limited conditions

Similarly, *C. curvatus* cells were also cultivated on acetate continuously under nutrient-limited conditions in a three-L stirred-tank bioreactor at dilution rates ranged from 0.01 to 0.11 h^−1^, and the results are shown in Table 3. Clearly, lipid content increased as the dilution rate decreased. The minimal lipid content was only 14.8 % at a dilution rate of 0.11 h^−1^, while the maximal lipid content of 66.4 % was obtained at a dilution rate of 0.01 h^−1^. The results indicated that *C. curvatus* favored lipid biosynthesis at lower dilution rates. Interestingly, lipid content and lipid yield under nitrogen-limited conditions were always lower than those under nitrogen-rich conditions at the same dilution rate (Table 3 vs Table 2). In fact, the presence of more yeast extract and peptone in the nitrogen-limited acetate medium might promote cell proliferation rather than lipid biosynthesis. As a result, the consumed C/N ratio remained low and disfavored higher lipid contents [34]. The lipid productivity increased when the dilution rate increased from 0.01 to 0.04 h^−1^, and then dropped when the dilution rate increased beyond 0.06 h^−1^ (Table 3). The highest lipid productivity was 1.32 g/L/d at the dilution rate of 0.04 h^−1^, which was higher than those under nitrogen-rich conditions.

**Table 3:** Results of continuous cultivation of *C. curvatus* at various dilution rates under nitrogen-limited conditions

*D* (h^−1^)	Acetate consumption (g/L)	Residual acetate (g/L)	Cell mass (g/L)	Non-lipid cell mass (g/L)	Lipid (g/L)	Lipid content (%)	Lipid yield (g/g)	Lipid productivity (g/L/d)
0.01	27.77 ± 0.17	5.09 ± 0.17	5.05 ± 0.13	1.70 ± 0.08	3.35 ± 0.06	66.40 ± 0.71	0.12 ± 0.00	0.80 ± 0.06
0.02	18.70 ± 0.54	14.15 ± 0.60	3.70 ± 0.09	1.41 ± 0.05	2.29 ± 0.04	61.92 ± 0.47	0.12 ± 0.00	1.10 ± 0.04
0.04	11.17 ± 0.52	21.69 ± 0.44	2.71 ± 0.05	1.34 ± 0.06	1.38 ± 0.01	50.72 ± 1.20	0.12 ± 0.01	1.32 ± 0.01
0.06	6.30 ± 0.51	26.56 ± 0.51	1.60 ± 0.04	0.97 ± 0.04	0.63 ± 0.01	39.30 ± 0.66	0.10 ± 0.01	0.91 ± 0.01
0.08	4.66 ± 0.46	28.20 ± 0.52	1.24 ± 0.08	0.92 ± 0.05	0.33 ± 0.04	26.26 ± 1.21	0.07 ± 0.01	0.63 ± 0.04
0.11	3.39 ± 0.61	29.47 ± 0.61	0.77 ± 0.03	0.66 ± 0.03	0.12 ± 0.01	14.87 ± 0.41	0.03 ± 0.01	0.32 ± 0.01

**Fig. 4:**
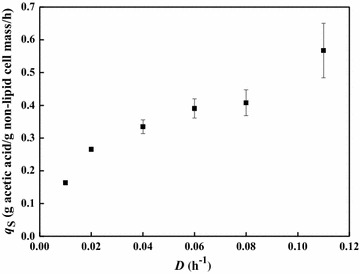
Steady-state specific acetic acid consumption rates of *C. curvatus* at different dilution rates under nitrogen-limited conditions. *Error bars* mean ± standard deviation of four samples

**Fig. 5:**
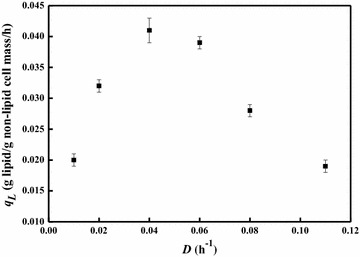
Steady-state specific lipid formation rates of *C. curvatus* at different dilution rates under nitrogen-limited conditions. *Error bars* mean ± standard deviation of four samples

As shown in Fig. 4, the specific acetate consumption rate increased along with the dilution rate. The maximal specific acetate consumption rate reached 0.57 g/g non-lipid cell mass/h at a dilution rate of 0.11 h^−1^. The specific lipid formation rate increased when the dilution rate increased from 0.01 to 0.04 h^−1^, albeit the lipid content decreased from 66.4 to 50.7 %. However, the specific lipid formation rate dropped when the dilution rate went beyond 0.06 h^−1^ (Fig. 5). Similar trends between the specific lipid formation rate and the dilution rate have been found for other oleaginous yeasts growing on glucose [24, 35]. The maximal specific lipid formation rate of 0.041 g/g non-lipid cell mass/h was observed at the dilution rate of 0.04 h^−1^, which was obviously lower than that under nitrogen-rich conditions. However, the result was 2.5-fold higher than that obtained by *Candida curvata* on glucose under nitrogen-limited conditions at the same dilution rate [36].

### Lipid production on corn stover hydrolysates containing acetate

**Fig. 6:**
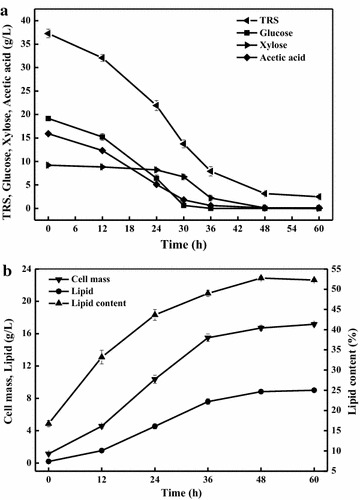
Results of lipid production by *C. curvatus* on corn stover hydrolysates containing acetate. **a** Profiles of substrates consumption. **b** Profiles of cell growth and lipid accumulation. *Error bars* mean ± standard deviation of three samples

To further demonstrate the capacity of lipid production by *C. curvatus* on acetate, we did enzymatic hydrolysis of alkaline-pretreated corn stover in an acetate buffer and generated the hydrolysates containing 19.2 g/L glucose, 9.2 g/L xylose and 15.9 g/L acetate. The hydrolysates were used to culture *C. curvatus* at pH 7.0 for 60 h, and the results are shown in Fig. 6. It was found that acetate was assimilated simultaneously with glucose, and that glucose, xylose and acetate were all consumed after 60 h (Fig. 6a). The residual glucose and acetate were both below 1.0 g/L within 36 h. Cell mass, lipid titre and lipid content increased over time (Fig. 6b). Cell mass, lipid titre, lipid content, lipid yield and lipid productivity were 17.2, 9.0 g/L, 52.3 %, 0.18 g/g substrate and 3.6 g/L/d, respectively. An overall lipid titre of 9.0 g/L is an indication that acetate indeed contributed substantially for lipid synthesis, as lipid yield would reach an unrealistic value of 0.32 g/g sugar. Furthermore, the lipid productivity was also several folds higher than those using acetate alone as substrate, suggesting a useful strategy to improve the rate of lipid production on acetate by addition of sugar [37]. Overall, our results suggest that acetate presented in biomass hydrolysates is effective carbon source for microbial lipid production.

## Conclusions

Acetate can be converted into microbial lipids by several oleaginous yeasts. Continuous conversion of acetate into lipids was promising under both nitrogen-rich and nitrogen-limited conditions by *C. curvatus*. The maximal specific lipid formation rate and lipid yield were higher under nitrogen-rich than nitrogen-limited conditions, which provided new opportunity for lipid overproduction from acetate-contained resources rich in nitrogen sources. Our results also suggest that biomass hydrolysates containing acetate are suitable for microbial lipid production. Further work should focus on elucidating the mechanisms of lipogenesis on acetate by oleaginous yeasts and developing more robust processes.

## Methods

### Strains and media

*C. curvatus* ATCC 20509 and *R. toruloides* ATCC 10788 were purchased from the American Type Culture Collection. *T. fermentans* CICC 1368 was purchased from the China Center of Industrial Culture Collection. *Lipomyces starkeyi* AS 2.1560, *Y. lipolytica* AS 2.1398, *T cutaneum* AS 2.571, *Rhodotorula glutinis* AS 2.107, *Rhodotorula mucilaginosa* AS 2.1515 and *Rhodotorula minuta* AS 2.277 were obtained from the China General Microbiological Culture Collection Center (CGMCC). *R. toruloides* Y4 was a derivative of *R. toruloides* AS 2.1389 obtained from CGMCC. The oleaginous yeasts were stored at 4 °C and propagated every 2 weeks on yeast peptone dextrose (YPD) agar slants (yeast extract 10 g/L, peptone 10 g/L, glucose 20 g/L, agar 15 g/L, pH 6.0). Yeasts inocula were prepared from YPD liquid medium (yeast extract 10 g/L, peptone 10 g/L, glucose 20 g/L, pH 6.0).

Acetate assimilation medium (g/L): Acetic acid 30, NH_4_Cl 1.07, H_3_PO_4_ 0.58, KCl 0.15, Na_2_SO_4_ 1.2, CaCl_2_ 0.22, MgCl_2_·6H_2_O 0.41, FeCl_2_·4H_2_O 0.0004 and vitamin stock solution 10 mL/L. Initial pH was brought to 7.0 by adding an appropriate amount of solid NaOH. The carbon-to-nitrogen (C/N) molar ratio of the medium was 50.

Nitrogen-rich acetate medium (g/L): Acetic acid 5, NH_4_Cl 5, yeast extract 0.1, peptone 0.1, H_3_PO_4_ 0.58, KCl 0.15, Na_2_SO_4_ 1.2, CaCl_2_ 0.22, MgCl_2_·6H_2_O 0.41, FeCl_2_·4H_2_O 0.0004 and vitamin stock solution 10 mL/L. Initial pH was brought to 7.0 by adding an appropriate amount of solid NaOH. This medium had a C/N ratio of 1.76.

Nitrogen-limited acetate medium (g/L): Acetic acid 30, NH_4_Cl 1.07, yeast extract 0.5, peptone 0.5, H_3_PO_4_ 0.58, KCl 0.15, Na_2_SO_4_ 1.2, CaCl_2_ 0.22, MgCl_2_·6H_2_O 0.41, FeCl_2_·4H_2_O 0.0004 and vitamin stock solution 10 mL/L. Initial pH was brought to 7.0 by adding an appropriate amount of solid NaOH. The medium had a C/N ratio of 35.5.

Vitamin stock solution (mg/L): thiamine hydrochloride 50, riboflavin 50, nicotinic acid 50, pantothenic acid 50, pyridoxine hydrochloride 10, biotin 20, folic acid 20, 4-aminobenzoic acid 50, cyanocobalamin 50, thioctic acid 50. It was filtered through 0.22 μm microporous membrane before added to the sterilized media.

Yeast extract (containing 3 % [wt/wt] ammonium-N and 9.0 % [wt/wt] total nitrogen) and peptone (animal-tissue based containing 3 % [wt/wt] ammonium-N and 14.5 % [wt/wt] total nitrogen) were obtained from Aoboxing Biotech. Co. Ltd. (Beijing, China). Antifoam 204, a mixture of organic polyether dispersions, was purchased from Sigma. Other reagents used were analytical grade and purchased from local company.

All the media were autoclaved at 121 °C for 18 min before use. Antifoam 204 0.1 % (w/v) was added for cultures in bioreactor.

### Batch culture

All pre-cultures were made in YPD medium at 30 °C, 200 rpm for 24 h unless otherwise specified. Cultures were initiated upon 45 mL of the acetate assimilation medium inoculated with 5 mL of pre-cultures in 250-mL unbaffled conical flasks. The cultures were held at 30 °C, 200 rpm for 72 h. Experiments were done in duplicates.

To 1.8 L of acetate assimilation medium was inoculated with 200 mL of pre-cultures, and the culture was performed at 30 °C, pH 7.0 in a 3-L stirred-tank bioreactor (Baoxing Biotechnology Inc., Shanghai, China). Agitation and aeration rate were set at 500 rpm and 0.8 vvm, respectively. The culture pH was maintained at 7.0 by automatic addition of 2 M H_2_SO_4_.

### Chemostat continuous culture

Pre-cultures 10 % (v/v) were inoculated to 1.8 L of acetate assimilation medium. The culture was initiated at 30 °C, pH 7.0, with agitation and aeration rate of 500 rpm and 0.8 vvm, respectively. The culture was changed to a continuous mode at 24 h with a specific dilution rate. Nitrogen-rich and nitrogen-limited chemostat continuous cultures were performed with a working volume of 1.85 L at 30 °C, pH 7.0. Agitation and aeration rate were maintained at 500 rpm and 0.8 vvm, respectively. Feeding medium and deformer were pumped into the bioreactor by a peristaltic pump (BT100-2J, Baoding Longer Precision Pump Co., Ltd). The chemostat were maintained at least four complete volume changes before sampling. It was assumed that steady states reached when cell and substrate concentrations changed less than 5 % within 12 h, while dissolved oxygen (dO_2_) output was used as the indicator for any perturbation [38]. Four samples for each steady state were collected at 4-h interval for analysis through the outlet port. Dilution rates were set ranging from 0.04 to 0.14 h^−1^ for nitrogen-rich culture and 0.01 to 0.11 h^−1^ for nitrogen-limited culture. The culture was shifted from the steady state at a higher dilution rate to the steady state at a lower dilution rate.

### Lipid production on corn stover hydrolysates containing acetate

Corn stover was alkaline-pretreated according to a published procedure and hydrolyzed using enzymes as described [29]. Briefly, the pretreated corn stover was loaded at 5 % (w/v) solid loading in 0.3 M acetate buffer (pH 4.8) and hydrolyzed at 50 °C for 48 h in the presence of 20 FPU cellulase, 40 CBU β-glucosidase and 5 mg xylanase per gram regenerated corn stover. The hydrolysates were boiled for 5 min, centrifuged and supplemented with 2 g/L (NH_4_)_2_SO_4_. The pH of the hydrolysates was adjusted to 7.0 before sterilization.

Cultures were initiated upon 45 mL of the hydrolysates inoculated with 5 mL of pre-cultures in 250-mL unbaffled conical flasks. The cultures were held at 30 °C, 200 rpm for 60 h. Cultivation pH was adjusted to 7.0 in 12-h time intervals. Experiments were done in triplicates.

### Analytical method

Glucose was determined using an SBA-40E glucose analyzer (Shandong Academy of Sciences, Jinan, China). Total reducing sugars (TRS) were quantified according to the 2, 4-dinitrosalicylate method with glucose as standard [39]. Xylose and acetate were measured by K-XYLOSE and K-ACETAF assay kit, respectively, from Megazyme [40].

Cell mass was determined gravimetrically after drying cells from certain volume of the culture broth at 105 °C overnight. Non-lipid cell mass was calculated after subtraction of lipids extracted from cell mass.

Lipid extraction was performed according to a published procedure [41]. Lipid content was expressed as gram lipid per gram dry cell weight. Lipid yield was calculated as gram lipid per gram carbon source consumed. The fatty acid compositional profiles of lipid samples were determined using a 7890F gas chromatography instrument after transmethylation according to a published procedure [26].

Acetate concentration (*C*_S_) and lipid concentration (*C*_L_) were constant within bioreactor at steady state. So, the specific substrate uptake rate (*q*_S_, g/g non-lipid cell mass/h) and the specific lipid formation rate (*q*_L_, g/g non-lipid cell mass/h) were calculated according to formulas (1) and (2), respectively.

1 $$q_{\text{S}} {\, =\, } \frac{{(C_{{{\text{S}}_{ 0} }} - C_{\text{S}} )}}{{C_{\text{Non-lipid cell mass}} }} \times D$$

2 $$q_{\text{L}} {\, =\, } \frac{{(C_{\text{L}} - C_{{{\text{L}}_{ 0} }} )}}{{C_{\text{Non-lipid cell mass}} }} \times D$$

$C_{{{\text{S}}_{ 0} }}$ is the acetate concentration of the solution used to feed the bioreactor, g/L; $C_{{{\text{L}}_{ 0} }}$ is the lipid concentration of the cells used to inoculate the bioreactor and equal to zero, g/L.

## Abbreviations

*C*: concentration
CBU: cellobiase unit
C/N: carbon-to-nitrogen
*D*: dilution rate
DCW: dry cell weight
FPU: filter paper unit
p*K*a: the negative logarithm of dissociation constant
*q*_S_: the specific acetate uptake rate
*q*_L_: the specific lipid formation rate
TRS: total reducing sugars
VFAs: volatile fatty acids
YPD: yeast peptone dextrose
